# Subunits of human condensins are potential therapeutic targets for cancers

**DOI:** 10.1186/s13008-018-0035-3

**Published:** 2018-02-20

**Authors:** Hong-Zhen Wang, Si-Han Yang, Gui-Ying Li, Xudong Cao

**Affiliations:** 1 School of Life Sciences, Jilin Normal University, Siping, 136000 P. R. China; 2 Key Laboratory for Molecular Enzymology and Engineering of The Ministry of Education, School of Life Sciences, Jilin University, Changchun, 130012 P. R. China; 3 Department of Chemical and Biological Engineering, University of Ottawa, Ottawa, K1N 6N5 Canada

**Keywords:** Human condensins, Subunit of human condensins, Cancer, Therapeutic target

## Abstract

The main role of condensins is to regulate chromosome condensation and segregation during cell cycles. Recently, it has been suggested in the literatures that subunits of condensin I and condensin II are involved in some human cancers. This paper will first briefly discuss discoveries of human condensins, their components and structures, and their multiple cellular functions. This will be followed by reviews of most recent studies on subunits of human condensins and their dysregulations or mutations in human cancers. It can be concluded that many of these subunits have potentials to be novel targets for cancer therapies. However, hCAP-D2, a subunit of human condensin I, has not been directly documented to be associated with any human cancers to date. This review hypothesizes that hCAP-D2 can also be a potential therapeutic target for human cancers, and therefore that all subunits of human condensins are potential therapeutic targets for human cancers.

**Electronic supplementary material:**

The online version of this article (10.1186/s13008-018-0035-3) contains supplementary material, which is available to authorized users.

## Introduction

In the past 20 years, one of the major breakthroughs in chromosome biology is the discovery of condensins [1]. While the main role of condensins is to regulate chromosome condensation and segregation during cell cycles [2], it is also known that condensins play roles in checkpoint, spindle assembly, and kinetochore organization [3–6]. To date, emerging evidence also suggests that condensins play important roles in mitosis, meiosis and interphase [2].

There are two kinds of condensins in human cells, known as condensin I and condensin II [7, 8]. Recently, it has been suggested in the literatures that subunits of condensin I and condensin II are involved in human cancers. This paper will briefly discuss discoveries of human condensins, their components and structures, and their multiple cellular functions. This will set the stage to review most recent studies on subunits of human condensins and their dysregulations or mutations in human cancers. It can be concluded that many of these subunits have potentials to be novel targets for cancer therapies except hCAP-D2—a subunit of human condensin I—which has not been directly documented to be associated with any human cancers to date. In this report, we hypothesize that hCAP-D2 can also be a potential therapeutic target for human cancers, and further studies should be focused on hCAP-D2 and on its potential role as a therapeutic target for human cancers.

## Discoveries of human condensins

Initial discoveries of human condensin was on the basis of studies on fog condensins. In 1994, a heterodimer XCAP-C/XCAP-E was purified from mitotic extracts prepared from *Xenopus* eggs by Hirano and Mitchison [9]. It was later established that the XCAP-C belonged to SMC4 protein subfamily and that XCAP-E belonged to SMC2 protein subfamily [10]. Subsequently a 13S pentameric complex containing XCAP-C/XCAP-E and three non-SMC subunits (i.e. XCAP-D2, XCAP-H and XCAP-G) was characterized and designated as “condensin” by the Hirano Lab in 1997 [1]. In 1998, an hCAP-C/hCAP-E complex, the human ortholog of XCAP-C/XCAP-E complex, was discovered to be responsible for mitotic chromosome condensation [11], and a 155-kDa protein interacting with hCAP-C/hCAP-E complex—termed condensation-related SMC-associated protein 1 (CNAP1)—was identified [12]. The CNAP1 was thought to be homologous to XCAP-D2 (also termed as Eg7) and later proven to be hCAP-D2 [13–15]. The discovery of CNAP1 indicated that there was a human protein complex that contained hCAP-C, hCAP-E, and hCAP-D2 in the HeLa nuclear extracts. It was unclear, however, whether the complex also contained hCAP-G and hCAP-H at that time [12]. In 2001, Kimura and colleagues [7] for the first time purified a pentameric human condensin I complex from HeLa nuclear extracts; about 2 years later, Ono et al. [8] discovered a second condensin complex—condensin II—in HeLa nuclear extracts.

## Components of human condensins

**Fig. 1:**
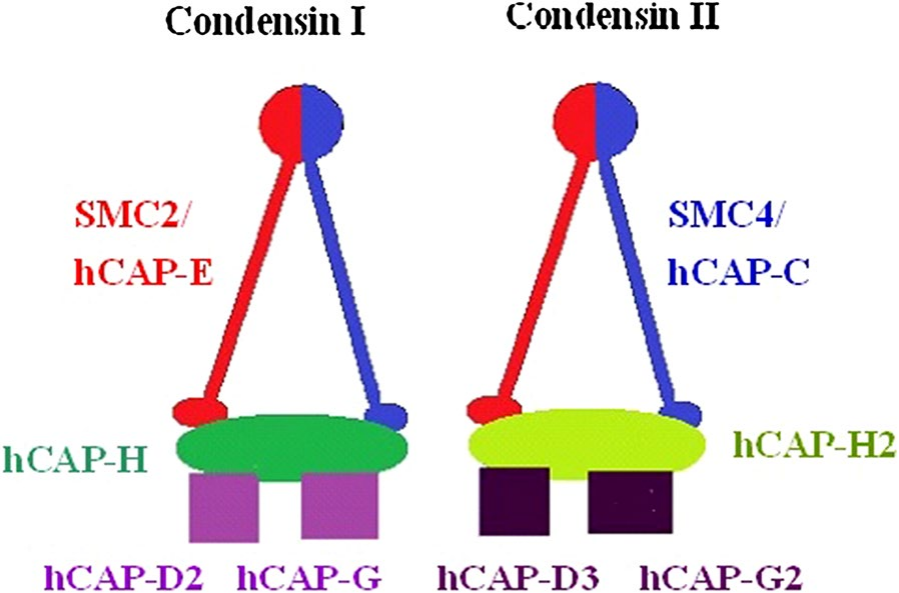
Structure and components of human condensin complex. The human condensin I and condensin II share the same heterodimer of SMC2 (hCAP-E) and SMC4 (hCAP-C). They have different sets of non-SMC subunits (hCAP-D2, hCAP-H, hCAP-G for condensin I and hCAP-D3, hCAP-H2, hCAP-G2 for condensin II)

Both of human condensins—human condensin I and human condensin II—are pentameric complexes consisting of shared core SMC2/SMC4 heterodimer and different sets of three-accessory non-SMC subunits. The shared core SMC2/SMC4 heterodimer in human cells is also known as hCAP-E/hCAP-C heterodimer [11]. In contrast, the three non-SMC subunits in human condensins are hCAP-D2, hCAP-H, and hCAP-G for condensin I and hCAP-D3, hCAP-H2, and hCAP-G2 for condensin II, as shown in Fig. 1.

Each subunit of human condensins is highly conserved in various organisms ranging from yeast to mammals [16]. The shared core subunit hCAP-E belongs to SMC2 protein subfamily and hCAP-C belongs to SMC4 protein subfamily [17]. One of the non-SMC subunits of each of the human condensins, hCAP-H in condensin I and hCAP-H2 in condensin II, belong to a kleisin family of proteins [18, 19]. A kleisin subunit is composed of conserved N- and C-terminal globular domains separated by a variable linker region in different organisms [18]. The other two non-SMC subunits of each of the human condensins, hCAP-D2, hCAP-G in condensin I and hCAp-D3, hCAP-G2 in condensin II, share a structural motif called HEAT repeats, belonging to HEAT repeat proteins [20, 21]. The HEAT repeats are repetitive arrays of short amphiphilic α-helices. The name “HEAT” comes from four proteins in which this repeat structure is found: Huntingtin, elongation factor 3 (EF3), protein phosphatase 2A (PP2A), and the signaling kinase TOR1 [22]. In short, each complex of human condensins contains two SMC core subunits, i.e. SMC2/hCAP-E and SMC4/hCAP-C, a kleisin subunit, i.e. hCAP-H or hCAP-H2, and two HEAT repeat proteins, i.e. hCAP-D2 and hCAP-G for condensin I as well as hCAP-D3 and hCAP-G2 for condensin II.

## Structures of human condensins

The shared SMC proteins of human condensins, hCAP-C and hCAP-E, can form a heterodimeric complex in HeLa nuclear extracts [11]. The primary structure of SMC proteins consists of five distinct domains. They are an N- and a C-terminal highly conserved domain (corresponding to the Walker A and B related segments of an ATPase), a central moderately conserved hinge domain flanked by two long coiled arms. The N- and C-terminal arms can form an intramolecular antiparallel coiled-coil that keeps the two terminal ATPase segments spatially together [23]. Each of the SMC subunits can self-fold intramolecularly and forms a rod-like structure with a globular ATPase “head” domain at one end and a “hinge” domain at the other. The central hinge domain is responsible for heterodimerization with the equivalent hinge domain of another SMC protein. Consequently, SMC2 and SMC4 form a heterodimer that adopts a V-shaped structure mediated by hinge–hinge interaction [24, 25]. The hinge domain of human condensin SMC2 subunit with short coils has been cloned [26]; recently the crystal structure of human condensin SMC hinge heterodimer with about 30 residues has been determinated [27]. It is interesting to mention that yeast condensin SMC2/SMC4 heterodimer has been shown to be flexible and can adopt various architectures such as O- and B-shaped conformations [28]. Therefore, it is likely that human condensin hCAP-C/hCAP-E heterodimer can also adopt conformations that are similar to their yeast counterparts.

It is known that the three non-SMC subunits of condensin I can form an 11S subcomplex by association with one another in Xenopus [29]. It is shown that the SMC2/SMC4 heterodimer further interacts with the three non-SMC subunits at their heads to form a lollipop-like closed-ring structure of human condensin I, as revealed by electron-microscopy [24]. In 2007, Onn et al. [21] successfully reconstituted a similar ~ 11S subcomplex from recombinant three non-SMC subunits of human condensin I and condensin II, respectively. The researchers also showed that both human condensin I and condensin II had a pseudo-symmetrical structure, in which the N-terminal half of kleisin (hCAP-H in condensin I or hCAP-H2 in condensin II) linked the first HEAT subunit (hCAP-D2 in condensin I or hCAP-D3 in condensin II) to hCAP-E, whereas its C-terminal linked the second HEAT subunit (hCAP-G in condensin I or hCAP-G2 in condensin II) to hCAP-C. Direct interactions between the hCAP-C/hCAP-E heterodimer and the HEAT subunits were not detectable. This indicates that the kleisin subunit acts as the connector in holocomplex assembly, as shown in Fig. 1.

## Multiple cellular functions and regulations of human condensins in mitosis

Increasing evidence shows that condensins play multiple roles in mitosis, meiosis and interphase in different organisms [2, 16, 30–33]. However, multiple functions and regulations of human condensins are reported mainly in mitosis and interphase.

Both human condensin I and condensin II are involved in mitotic chromosome condensation and show different distributions along the axis of chromosomes assembled both in vivo and in vitro [7, 8]. Chromatids were a little swollen and the anti-hCAP-E signals were spanned the whole width of the chromatids after depletion of condensin I whereas the hCAP-E signals remained concentrated in the central core of the chromatids after depletion of condensin II [8]. Hirota et al. [34] reveal that human condensin I and II sequentially associate with chromosomes. Condensin II associates with chromatin, which is needed for chromosome condensation in early prophase. Condensin I is in cytoplasm at that time and appears to be dispensable at this stage. Only after nuclear envelope breakdown can condensin I interact with chromosomes. In contrast, condensin I is required for subsequent dissociation of cohesin from chromosome arms, chromosome shortening and normal timing of progression through prometaphase and metaphase, whereas condensin II are dispensable for these processes. Ono et al. [35] show that human condensin I and condensin II alternate along the axis of metaphase chromatids and that depletion of condensin subunits affects kinetochore structure and function. Subsequently, human condensin I has been shown to be accumulated at transcription start site [36]. Recently, Ono et al. [37] further demonstrate that the recovery of chromatin shapes and the reorganization of axes are less sensitive to depletion of condensin I or topoisomerase IIα, but highly sensitive to depletion of condensin II with a set of two-step protocols for reversible assembly of chromosome structure within a cell. The differences in the timing of chromosome binding and mutant phenotypes of depletion strongly suggest that human condensin I and II have fundamentally distinct mitotic functions.

Secondly, human condensins play pivotal roles in forming structure of centromeres and affect chromosome segregation. Gerlich et al. [38] showed that human condensin I depletion impaired mechanical stability of centromeres. Human condensin I showed a two-step dynamic binding. After nuclear envelope breakdown, human condensin I rapidly associated with mitotic chromosomes then remained constant from prometaphase to late metaphase. Just before anaphase onset, chromatin bound human condensin I increased again before it dissociated from chromosomes in late anaphase. Human condensin I-depleted cells show an increased interkinetochore distance. It seems that human condensin I have a role to lock already condensed chromatin by human condensin II in a rigid state for stable spindle attachment. Consist with this, Takahashi et al. [39] found that KIF4A associates with human condensin I but not with human condensin II in mitosis. Without binding to KIF4A, human condensin I fails to confer rigidity to centromeres. When human condensins are depleted, centromeres are extremely stretched and the corresponding kinetochores suffer merotelic attachments. Ultimately, chromosomes enter anaphase with improperly structured kinetochores and chromosome bridges that break during or before cytokinesis [40]. Likewise, Zhai et al. [41] observed chromatin bridges between daughter cells and multiple nuclei in single cells when human condensins are knocked down. Similarly, condensin dysfunction in human cells induces nonrandom chromosomal breaks in anaphase with specifically destabilized in both repeated and nonrepeated parts of the genome [42].

Thirdly, there are many reports on phosphorylations of human condensin subunits by different kinase and possible mechanisms for activation and regulation of human condensins during mitosis. For example, in vitro studies indicated that cyclin-dependent kinase 1 (Cdk1)-mediated phosphorylation of the non-SMC subunit set is required for chromosomal localization of human condensin I and stimulation of its supercoiling activity [7]. Cdk1-mediated phosphorylation of condensin I is the sole mitosis specific modification required for chromatids reconstitution in vitro by a minimum set of purified factors [43]. Takemoto et al. [44] further confirmed that the levels of condensin I subunits were constant by quantitative immunoblot analysis and human condensin I is phosphorylated throughout the cell cycle. The phosphorylation of non-SMC subunits correlates with the chromosomal targeting of condensin I and stimulation of its biochemical activity. Blank et al. [45] shows that DNA damage in cells with a compromised p53-mediated G2/M checkpoint triggers the activation of Cdk1, activation and chromatin loading of human condensin I, and uneven chromatin condensation (UCC) followed by the appearance of multimicronucleated cells. Similarly, Mitotic chromosome condensation in prophase depends on Cdk1-mediated phosphorylation of hCAP-D3. Mitotic phosphorylation of condensin II depends on both Cdk1 and Plk1. Thr1415 of the hCAP-D3 as a Cdk1 phosphorylation site is required for the polo kinase Plk1 (polo-like kinase 1) to localize to chromosome axes and further phosphorylation of the condensin II complex by Plk1 [46]. Activation of condensin II by Cdk1-mediated phosphorylation is based on that chromosome condensation takes place before the nuclear envelope breakdown in cells depleted of Plk1 activity [47]. Recently, Kagami et al. [48] shows Plk1 phosphorylation of hCAP-H2 at Ser288 also triggers chromosome condensation at the early phase of mitosis. But the molecular mechanism in their report is different from that of mitotic chromosome condensation caused by phosphorylation of hCAP-D3 [46]. The protein levels of hCAP-H2 are increased in mitosis by a Plk1 kinase activity-dependent manner. Inhibition of Plk1 induces cell-division cycle protein 20 (Cdc20)-mediated degradation of hCAP-H2 [48].

Apart from Cdk1 and Plk1, there are other kinds of kinase involved in phosphorylation and regulation of human condensins. For example, Mps1, Aurora B, Ark1 and polo-like kinases are also reported to phosphorylate and regulate human condensins [49–52]. Wike et al. [53] also show that Aurora-A mediated phosphorylation of histone H3 threonine 118 can indirectly reduce condensin I occupancy on chromatin via its influence on chromosome packaging. Two kinds of kinase can operate to regulate function of human condensin I. For example, recently Poonperm et al. [54] reported that aurora B activity facilitates the targeting of KIF4 and condensin I to the chromosome, whereas Plk1 activity promotes the dissociation of these proteins from the chromosome. It is interesting to explore how different mitotic kinase subsequently cooperates to regulate function of human condensin I and condensin II.

Independent role of Cdk1, human condensins plays a role in chromosome condensation has also been proposed; however, the mechanism of its involvement is unclear [55].

## Multiple cellular functions and regulations of human condensins in interphase

During interphase, human condensin I is predominantly cytosolic and human condensin II is primarily nuclear [8, 34]. Human condensin II is thought to be the major player in nucleus during interphase. However, it has been reported that a small amount of human condensin I persists within the nucleus during interphase [12, 56]. In addition to their mitotic functions, human condensins have been implicated in chromosome organization and gene expression during interphase.

During interphase, the distinct punctate nucleolar localization of hCAP-H, hCAP-C, and hCAP-E suggests that the human condensins play a role in the conformation and function of rDNA [56]. Consistent with this, Huang et al. [57] discovered that human condensins and CCCTC-binding factor (CTCF) bind to specific regions of human rDNA in a competitive manner. Human condensins negatively regulate CTCF-mediated rRNA gene.

Likewise, colocalization and interaction of DNA ligase IV and hCAP-E in the interphase nucleus suggests that human condensins also play a role in nuclear architecture [58]. For example, human condensin I interacts with the PARP-1-XRCC1 complex and plays a role in the repair of DNA single-strand breaks. This process is mediated by PARP1 through its interaction with the chromosome-targeting domain of hCAP-D2 [59, 60]. George et al. [61] show that depletion of the hCAP-H2, hCAP-D3 and hCAP-E subunits of human condensin II leads to dramatic changes of nuclear shape and increased nuclear size. This is consistent with the model that condensin mediated chromatin compaction contributes to the prestressed condensed state of the interphase nucleus.

Chromosome condensation is also important during interphase if it occurs at transcriptionally silent, heterochromatic regions such as centromeres [62]. For example, when human condensins are depleted, centromeres with impaired kinetochores are extremely stretched [40]. Similarly, chromatin condensation via the human condensin II is required for proper T cell development and maintenance of the quiescent state [63]. During early G1, Barnhart-Dailey et al. [64] observed that human conden-sin II localization to centromeres is dependent on Holliday junction recognition protein (HJURP). HJURP induces decondensation of a noncentromeric LacO array. Human condensin II can counteract the induced chromatin decondensation by HJURP and is required for new CENP-A deposition at the centromere. During S phase, a subfraction of human condensin II starts to associate with chromatin and compact replicated regions of chromosomes. It suggests that human condensin II prepare early for sister chromatids resolution during S phase and proper condensation and segregation in mitosis [65]. In G2 phase, cells from hMCPH1 (also known as microcephalin or BRIT1) patients were found to display premature chromosome condensation (PCC) [66, 67]. The PCC phenotype can be rescued by depletion of specific subunits of human condensin II [67, 68]. Yamashita et al. [69] further demonstrate that hMCPH1 acts as a composite modulator of human condensin II and activity of human condensin II has been inhibited by hMCPH1 during G2 phase to prevent PCC.

In addition to involving in chromatin condensation during interphase, it is notable that in a previous report, human condensin II associating with hMCPH1 plays a role in homologous recombination repair [68].

Other than this, interaction of human condensin II with the gamma-interferon activated inhibitor of translation (GAIT) subunits can restrict L1 retrotransposition through inhibition of L1 transcription and translation. The novel mechanism of L1 repression contributes to the maintenance of genome stability in somatic cells [70].

There are multiple regulations of human condensins in interphase. For example, human condensin subunits were co-immunoprecipitated with the DNA methyltransferase DNMT3B (DNA methyltransferases 3B), which is known to associate with the histone deacetylase HDAC1. Thus, histone modification appears to play a general role in condensin targeting [71]. Consistent with this, hCAPD3 and hCAPG2 are reported to interact with H4K20me1 histone tails and support specific association between H4K20me1 and condensin II [72]. Recently, human condensin II is also anchored by TFIIIC and H3K4me3 and supports the expression of active dense gene clusters [73]. Additionally, Zhang et al. [74] report that the G2 damage checkpoint prevents stable recruitment of human condensin I and condensin II onto the chromatin. During G2 arrest, the inhibition of condensins recruitment to prevent chromosome compaction is mediated specifically by the Chk2 kinase and no need of an active Cdk1. Takemoto et al. [75] demonstrate CK2-dependent phosphorylation of human condensin I increases during interphase and decreases during mitosis. The negative regulatory mode for human condensin I may influence chromatin structure during interphase and mitosis.

Taken together, these results reveal multiple cellular functions and versatile regulations of human condensins during the interphase.

## Dysregulations or mutations of subunits of human condensins in cancers

In mitosis, it is critical for chromosomes to be properly segregated for the faithful transmission of genetic information. In interphase, chromosomes are relatively less compact and accessible to genetic and epigenetic information encoded by DNA and chromatin. As mentioned above, human condensins play so many important functions and regulations involved in chromosome architecture during mitosis and interphase. Since it is known that dysfunction of human condensin I and condensin II can cause defects in chromosome condensation and segregation, it is no surprise that dysregulations or mutations of subunits of human condensins involved in chromosomal instability. Chromosomal instability is a form of genomic instability and is a characteristic of many cancers.

Accumulating evidence suggest that many subunits of human condensin I and condensin II are involved in human cancers (see Additional file 1: Table S1), as will be discussed in the following text.

Firstly, SMC2 and SMC4 (i.e. hCAP-E and hCAP-C), the core subunits of human condensin I and condensin II, have been shown to be associated with some malignant tumors. In particular, SMC2 has been shown to be dysregulated or mutated in pyothorax-associated lymphoma (PAL), *MYCN*-amplified neuroblastoma, gastric and colorectal cancers [76–78]. In detail, a 117 bp deletion corresponding to exon 24 and a transition of the invariable G → A at position + 1 at the donor splice site of intron 24 within *hCAP*-*E* were detected in OPL-5 cell line and original tumor samples [76]. Expression of hCAP-E in OPL-5 with aberrant skipping of exon 24 in *hCAP*-*E* showed a reduction. OPL-5 occasionally showed the chromosome bridge in anaphase and telophase [76]. SMC2 expression is elevated in *MYCN*-amplified human neuroblastoma cells and down regulation of SMC2 induced DNA damage and apoptosis in human neuroblastoma cells [77]. SMC2 gene is exclusively altered by both frameshift mutation and loss of expression in gastric cancers and colorectal cancers with high microsatellite instability [78]. In addition, SMC2 has also been demonstrated to be one of two breast and ovarian cancer risk loci [79]. Treatment of WNT-activated intestinal tumor cells with SMC2 siRNA significantly reduced cell proliferation in nude mice when compared with untreated controls (p = 0.02) [80]. As a result, SMC2 has been studied as a new molecular therapeutic target for cancer treatment [77, 80].

Similarly, SMC4 has also been shown to dysfunction in PAL, breast cancer, hepatocellular carcinoma (HCC), colorectal cancer (CC), lung adenocarcinoma, prostate cancer and glioma [76, 81–87]. In detail, in pyothorax-associated lymphoma, missense mutations of the *hCAP*-*C* gene involve G2294A (Ala → Thr) in OPL-3 cell line and C1410T (Ala → Val) in OPL-7 cell line. OPL-7 showed down regulated expression of hCAP-C [76]. In breast cancer, SMC4 is down-regulated in cell lines that were paclitaxel-resistant but combination synergistic [81]. On the contrary, SMC4 mRNA and protein are highly expressed in HCC samples and cell lines compared to the normal [82]. SMC4 transcription and expression is negatively regulated by miR-219 in human hepatocellular carcinoma 97-H, HepG2 cell lines [83]. That is SMC4 upregulated in hepatocellular carcinoma. Similarly, SMC4 was upregulated in colorectal cancerous tissue and knockdown of SMC4 plays a suppressive role in the proliferation, cell cycle and apoptosis of colorectal cancer cells [84]. Jinushi et al. [85] demonstrated that higher miR-124-5p expression correlated with a higher overall survival of colorectal cancer patients by inhibited the expression of SMC4. Moreover, SMC4 is overexpressed in lung adenocarcinoma tissues and acts as an independent prognostic predictor. SMC4 silencing inhibits the proliferation and invasion of lung adenocarcinoma cells [86]. In prostate cancer, higher expression of SMC4 is significantly associated with the metastatic cascade [87]. Recently, SMC4 upregulation markedly promoted the glioma cell proliferation rate and migration and invasive capability in vitro and in vivo [88]. As a result, SMC4 has been suggested as a potentially novel therapeutic target for cancer therapy [82, 88].

Secondly, two non-SMC subunits of condensin I have been demonstrated to be dysregulated or mutated in human cancers, and therefore could be potential novel therapeutic targets. For example, NCAPH (i.e. hCAP-H) is upregulated in melanomas [89]. Recently, Yin et al. [90] found missense and depletion mutations of NCAPH in CC patients and showed that non-SMC condensin I complex subunit H (i.e. hCAP-H) is overexpressed in colorectal cancer(CC)cell lines in comparison with normal human cells. Therefore hCAP-H has been suggested as a new possible therapeutic target for CC. Likewise, NCAPG (i.e. hCAP-G) is also upregulated in melanomas [89]. Additionally, NCAPG is upregulated and miR-6500-3p is downregulated in pediatric high-grade gliomas, which are aggressive brain tumors affecting children. Liang et al. [91] identified that NCAPG is target of miR-6500-3p and 3′UTRs of NCAPG is directly targeted by miR-6500-3p. NCAPG knockdown caused cell cycle arrest in G1 phase and impaired cell proliferation. NCAPG plays an important role in the development and progression of HCC. NCAPG was overexpressed in HCC compared with the adjacent normal tissue (p < 0.001) [92]. Knockdown of NCAPG induces HCC cell mitosis and inhibits cell growth, proliferation and migration in vitro and tetracycline-inducible shRNA knockdown of NCAPG inhibits tumor growth of HCC cells in vivo [93]. Recently, NCAPG were negatively regulated by miR-145-3p and miR-145-3p was downregulated in prostate cancer cells [94]. That is NCAPG upregulated in prostate cancer. On the contrary, NCAPG was downregulated in multiple myeloma and acute myeloid leukemia [95]. Similarly, hCAP-G has also been suggested to be a promising prognostic marker and therapeutic target for HCC [92, 93].

Thirdly, all three non-SMC subunits of condensin II have been demonstrated to exhibit abnormal expressions in cancer cells and have been exploited as potential novel therapeutic targets for human cancers. For example, Lapointe et al. identified hCAP-D3 as a new tissue biomarker overexpressed in subtype-1 prostate cancer cells [96]. Another non-SMC condensin II complex subunit G2 (i.e. hCAP-G2) has also been found to be overexpressed in melanomas [89]. Interestingly, in a study by Shiheido et al. [97] the researchers report that Q15, an anilinoquinazoline derivative, is a binding partner for hCAP-G2, and that Q15 shows potent in vitro growth-inhibitory activities towards cancer cell lines derived from colorectal cancer, lung cancer and multiple myeloma (MM). This strongly suggests that hCAP-G2 is a promising therapeutic target for cancer therapies. In addition, simultaneously targeting hCAP-G2 and MIP-2A, another Q15-binding protein, has been demonstrated to be a promising strategy to develop antitumor drugs for intractable tumors [98]. In support of the above findings, most recent study by Zhan and colleagues [99] shows that hCAP-G2 is overexpressed in non-small cell lung cancer (NSCLC) patients and suggests the notion that the hCAP-G2 expression can be used as a prognostic biomarker in lung adenocarcinoma. The data from this study also argue for the potential application of hCAP-G2 as a promising therapeutic target for NSCLC patients [99]. Finally, using a large-scale genome-wide association study in patients (n > 5000), Law et al. [100] report that hCAP-H2, a non-SMC subunit of human condensin II, is a new risk locus for chronic lymphocytic leukemia (CLL) and multiple myeloma (MM).

Based on the discussion above, it is evident that other than hCAP-D2, all other subunits of both condensin I and condensin II have been shown to be dysregulated or mutated in human cancer cells. While there is no direct evidence to suggest the involvement of the hCAP-D2 subunit in human cancers, it is worthwhile to note that there is emerging evidence to suggest that hCAP-D2 can indeed interact with other two non-SMC subunits of condensin I, hCAP-G and hCAP-H, the abnormal expression of which are both identified as biomarkers for cancers and reported as potential targets for therapeutic treatments for cancers [90, 92]. For example, Watrin et al. [101] report that association of hCAP-H with mitotic chromosomes depends on the presence of hCAP-D2. In addition, Kinoshita et al. [102] show that mutant condensin I lacking either hCAP-D2 or hCAP-G produces abnormal chromosomes with highly characteristic defects. Because condensin I plays such a critical role in mitotic chromosome assembly and segregation from yeast to humans, dysfunction of either one subunit of condensin I can cause chromosome aberration and genomic instability. Chromosome aberration and genomic instability is a common feature for cancer cells.

Given that both hCAP-H and hCAP-G are dysregulated or mutated in human cancers and that hCAP-D2 has close associations with both, we believe that there is a very good chance for hCAP-D2 to be also involved in human carcinogenesis.

Finally, there are strong evidence to suggest good correlations between mouse and human in the conservative functions of condensins. For example, in an earlier work, Woodward et al. report that mutation to CAP-H2 (a murine orthologue to hCAP-H2) results in genomic instability and murine T-cell lymphomas [103]; approximately 1 year later, Law et al. [100] are successful in identifying hCAP-H2 as a new risk locus of human CLL. Interestingly, Cai et al. [104] recently report that Ncapd2 (a murine orthologue to hCAP-D2) is one of 79 DEGs that are differentially expressed in late carcinoma stage (week 12) during murine breast cancer progression. It is therefore would be interesting to investigate if hCAP-D2 is dysregulated or mutated in related human cancers.

In conclusion, dysfunction of hCAP-D2 can affect its interactions with the other two non-SMC subunits of condensin I and can cause genomic instability. Genomic instability, a hallmark of cancer associated with poor prognosis, tumor heterogeneity and the development of therapy resistance [105]. Additionally, similar to the situation of hCAP-H2, murine orthologue of hCAP-D2 has been demonstrated to be differentially expressed in murine cancers. It is therefore reasonable to hypothesize that hCAP-D2 may be involved in human carcinogenesis and that it can be a potential therapeutic target for human cancers. This hypothesis should be tested in future investigations. If this hypothesis is true, all subunits of human condensins can be potential therapeutic targets for human cancers.

## Additional file

**Additional file 1: Table S1.** Subunits of human condensin I and condensin II that are involved in cancers.

## Abbreviations

SMC: structural maintenance of chromosomes
XCAP: Xenopus chromosome associated polypeptides
hCAP: human chromosome associated protein
CNAP1: condensation-related SMC-associated protein 1
Cdk1: cyclin-dependent kinase 1
cdc2: cell division control protein kinase 2
UCC: uneven chromatin condensation
Plk1: polo-like kinase 1
cdc20: cell-division cycle protein 20
DNMT3B: DNA methyltransferases 3B
CTCF: CCCTC-binding factor
PARP-1: poly(ADP-ribose) polymerase-1
XRCC1: X-ray repair cross-complementing protein 1
HJURP: Holliday junction recognition protein
hMCPH1: also known as microcephalin or BRIT1
PCC: premature chromosome condensation
GAIT: the gamma-interferon activated inhibitor of translation
CHK2: checkpoint kinase 2
CK2: casein kinase 2 or casein kinase II
MYCN: n-myc
WNT: wingless/int1
PAL: pyothorax-associated lymphoma
siRNA: small interfering RNA
MIP-2A: MBP-1 interacting protein-2A
HCC: hepatocellular carcinoma
CC: colorectal cancer
NCAPH: non-SMC condensin I complex subunit H
NCAP-G: non I SMC condensin I complex subunit G
NCAPG2: non-SMC condensin II complex subunit G2
MIP-2A: MBP-1 interacting protein-2A
NSCLC: non-small cell lung cancer
CLL: chronic lymphocytic leukaemia
MM: multiple myeloma
DEG: differentially expressed gene
