# Supplementary material for: Subunits of human condensins are potential therapeutic targets for cancers
Source: Cell Div. 2018 Feb 20;13:2. doi: 10.1186/s13008-018-0035-3 (PMC5819170; doi:10.1186/s13008-018-0035-3)
Supplement: Supplementary file 1 — Additional file 1: Table S1. Subunits of human condensin I and condensin II that are involved in cancers. [file 13008_2018_35_MOESM1_ESM.docx]

ADDITIONAL TABLE 1

**Table S1 Subunits of human condensin I and condensin II that are involved in cancers**

| condensin subunit | | type of cancer | type of mutation / expression of a subunit | reference and publication year |
| --- | --- | --- | --- | --- |
| shared core SMC2/SMC4 heterodimer | SMC2 | pyothorax-associated lymphoma (PAL) | del exon 24,G1A (at the donor splice site of intron 24) (OPL5)/  underexpressed | [76]，2007 |
|  |  | *MYCN*-amplified neuroblastoma | overexpressed | [77], 2014 |
|  |  | gastric and colorectal cancers | frame shift mutation/ loss of expression | [78], 2014 |
|  |  | breast and ovarian cancer | mutation at the 9q31，5′-untranslated region of SMC2 | [79], 2016 |
|  |  | WNT-activated hyperplastic cells | overexpressed | [80], 2012 |
|  | SMC4 | pyothorax-associated lymphoma (PAL) | G2294A (OPL3),C1410T(OPL7)/underexpressed | [76], 2007 |
|  |  | breast cancer | underexpressed | [81], 2011 |
|  |  | HCC | overexpressed | [82,83], 2012 ,2014 |
|  |  | colorectal cancer (CC) | overexpressed | [84,85], 2014 |
|  |  | lung adenocarcinoma | overexpressed | [86]，2016 |
|  |  | prostate cancer | overexpressed | [87]，2016 |
|  |  | glioma | overexpressed | [88]，2017 |
| Non-SMC subunits of human condensin I | hCAP-D2 | not reported | not reported | not reported |
|  | hCAP-H | melanoma | overexpressed | [89]，2007 |
|  |  | CC | Missense, del  overexpressed | [90]，2017 |
|  | hCAP-G | melanoma | overexpressed | [89]，2007 |
|  |  | glioma | overexpressed | [91]，2016 |
|  |  | HCC | overexpressed | [92,93]，2017 |
|  |  | prostate cancer | overexpressed | [94]，2017 |
|  |  | myeloma and acute myeloid leukemia | underexpressed | [95]，2014 |
| Non-SMC subunits of human condensin II | hCAP-D3 | subtype-1 prostate cancer | overexpressed | [96]，2008 |
|  | hCAP-H2 | CLL, multiple myeloma (MM) | A50950076AT | [100]，2017 |
|  | h CAP-G2 | melanoma | overexpressed | [89]，2007 |
|  |  | CC, lung cancer, MM | overexpressed | [97,98]，2017 |
|  |  | lung adenocarcinoma | overexpressed | [99]，2017 |
